# Stakeholder Perspectives on Facilitators of and Barriers to the Implementation of Internet of Things–Equipped Powered Wheelchairs in Sweden: Qualitative Case Study

**DOI:** 10.2196/92414

**Published:** 2026-08-28

**Authors:** Johan Borg, Marie Elf, Anders Brantnell, Klas Palm

**Affiliations:** 1 School of Health and Welfare, Dalarna University, Högskolegatan 2, Falun, SE-79188, Sweden, +46 23778959; 2 Department of Civil and Industrial Engineering, Division of Industrial Engineering and Management, Uppsala University, Uppsala, Sweden; 3 Department of Women’s and Children’s Health, CIRCLE - Complex Intervention Research in Health and Care, Uppsala University, Uppsala, Sweden

**Keywords:** assistive products, assistive technology, implementation, Internet of Things, IoT, powered wheelchairs

## Abstract

**Background:**

The implementation of Internet of Things (IoT) technologies to proactively monitor the functioning of assistive products remains underexplored, particularly in public assistive technology services.

**Objective:**

This qualitative single-case study investigated how a public assistive technology provider in Sweden implemented IoT-equipped assistive products.

**Methods:**

The implementation process was mapped, and a thematic analysis of interviews with 6 participants representing key organizational roles was conducted.

**Results:**

The process comprised 9 major events involving 4 main stakeholders. The findings included 4 themes of facilitators and 5 themes of barriers, which were synthesized into three meta-themes: (1) system-level structures that enable and constrain implementation, (2) tensions between technological potential and the challenges of realizing it, and (3) relational and cultural dynamics that balance trust with caution.

**Conclusions:**

The findings underscore the importance of using a sociotechnical framework to guide the implementation of IoT in assistive technology services. Such a framework supports a holistic approach that integrates user needs, privacy considerations, and regulatory requirements, while drawing on leadership, technical expertise, and collaboration to address legal, organizational, technical, and user-related challenges. The implications for health care technology implementation are that organizations should adopt sociotechnical implementation strategies that account for legal, organizational, technical, and user-related considerations.

## Introduction

Assistive products that function poorly can limit users’ participation at home and in the community and may lead to reduced quality of life, injury, or pain [1-4]. Maintenance and repair services are therefore essential components of the provision of assistive products [5]. Using technologies such as the Internet of Things (IoT) to monitor use, condition, and status offers a proactive approach to identifying maintenance needs, preventing users from being stranded, and providing insights that can inform design improvements.

IoT refers to a network of uniquely identifiable objects connected to the internet, often equipped with sensing, actuation, and programming capabilities [6]. These capabilities allow data collection and remote adjustment of device states. In health care, IoT has been recognized as a tool for improving quality and efficiency, enabling real-time monitoring and enhanced coordination among devices, service users, and providers. Such connectivity can support more proactive and integrated care, potentially reducing system strain by lowering resource demands and workloads [7,8].

Breakdowns and repairs of powered wheelchairs are relatively common [2-4]. Applying IoT to assistive products like these enables remote monitoring (eg, overheating and location), optimization of individual settings (eg, speed, footrest angle), troubleshooting (eg, identifying damaged components), and maintenance planning (eg, detecting battery degradation). Thus, the IoT has the potential to improve the management and use of assistive products, and consequently, enhance users’ quality of life. However, evidence regarding the successful implementation of IoT in health care remains limited [9], and to our knowledge, no studies have examined the implementation of IoT-equipped assistive products in practice.

Successful IoT adoption in health care is challenging because it involves more than introducing new devices. It requires integrating new technologies into an existing network of actors, routines, and governance structures [10,11]. A sociotechnical perspective can help explain why this integration succeeds or fails by treating implementation as a dynamic interaction of factors across social, organizational, and technical dimensions that influence each other [9]. From this perspective, leadership is central for providing direction, legitimacy, and resources [12]. The involvement of critical actors within the organization can support local adaptation, workflow fit, and trust among users. Understanding how these dimensions influence each other is important for translating IoT’s potential into sustained use in health services [13].

The sociotechnical perspective has been applied in health care research such as the adoption of health IT [14], the usability of electronic health records [15], and the use of AI in health care [16]. Although these studies demonstrate the importance of studying health care technologies as sociotechnical systems, in which the implementation depends on interactions between technical and organizational factors [14-16], few studies have examined the actual implementation of IoT solutions in health care practice [9]. Moreover, a recent review of IoT adoption in health care did not identify any studies exploring the implementation process from a sociotechnical perspective, particularly regarding IoT-equipped assistive products within assistive technology services [9].

Furthermore, to our knowledge, no previous studies have specifically investigated the implementation of IoT-equipped assistive products in real-world health care settings. Consequently, there is insufficient understanding of how social, organizational, and technical factors interact during the implementation of IoT-equipped assistive products.

The objective of this study was to explore the implementation process of IoT-equipped powered wheelchairs within a public assistive technology provider in Sweden from a sociotechnical perspective. The study focused on the implementation journey from initiation through to the organizational decision to deploy the technology to end users.

Specifically, it addressed the following research questions:

1. What were the major events that shaped the implementation of IoT-equipped powered wheelchairs?
2. Who were the stakeholders involved in this process and what roles did they play?
3. What facilitators and barriers did stakeholders perceive as influencing the implementation of IoT-equipped powered wheelchairs?
4. How can the implementation of IoT-equipped powered wheelchairs be understood from a sociotechnical perspective?

## Methods

### Qualitative Approach and Theory

This study employed a qualitative single-case study design [17,18], using thematic analysis of semistructured interviews. The reporting is structured in compliance with the Standards for Reporting Qualitative Research [19].

**Figure 1.**
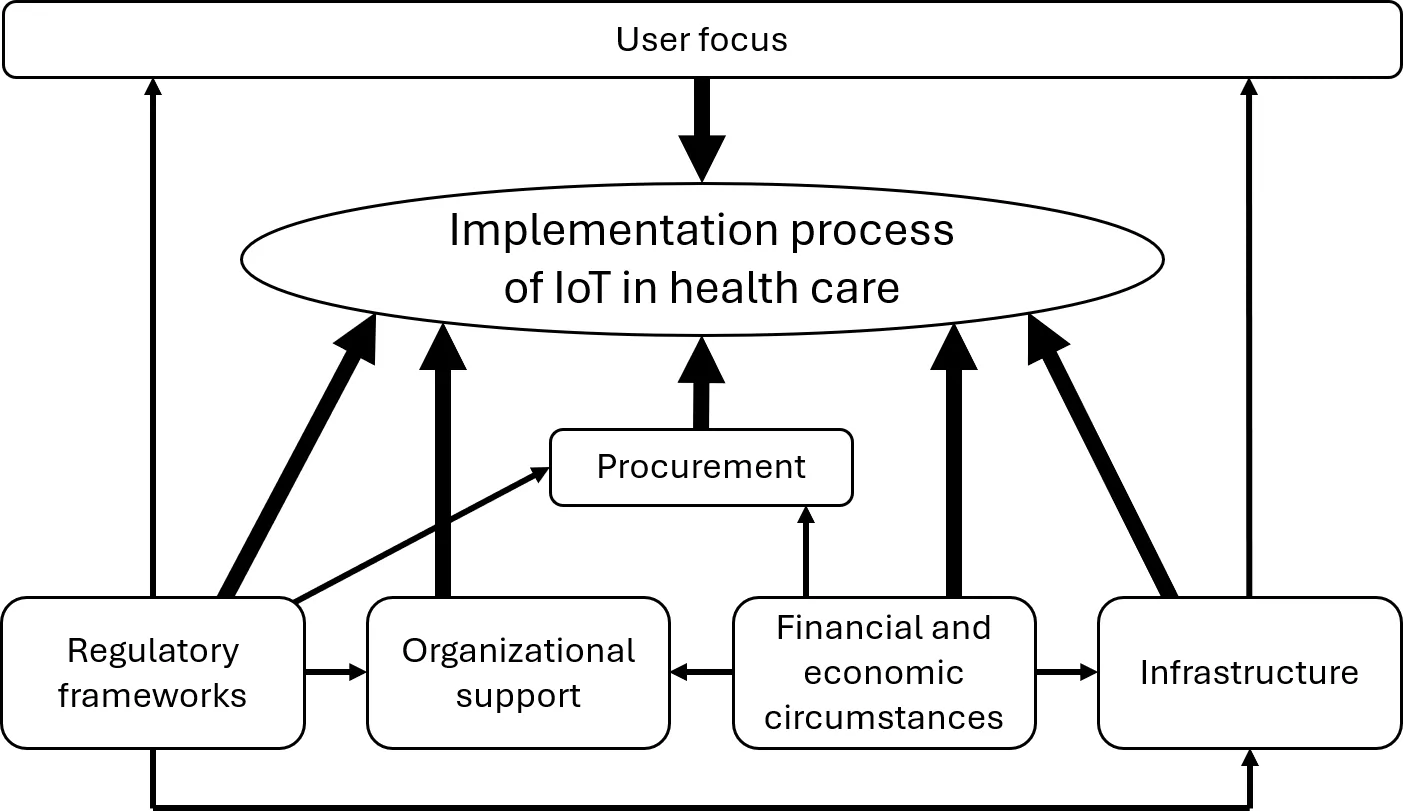
A sociotechnical systems model tailored to Internet of Things (IoT) implementation in health care, showing the most important factors that seem to influence the IoT implementation process in health care. The broader arrows represent a direct impact on the IoT implementation process in health care, while the narrower arrows represent an indirect impact, from one factor to another factor, which, in turn, affects the ability to implement IoT in health care (adapted from Palm et al [21]).

To provide a theoretical perspective and enhance the understanding of the adoption process, we drew on the sociotechnical framework developed by Davis et al [20] and later enhanced by Palm et al [21], as the original framework was found not to fully cover important dimensions in the implementation of IoT in health care [9]. The enhanced sociotechnical systems model is tailored to IoT implementation in health care and includes the most important factors that seem to influence the implementation process (Figure 1) [21].

### Researcher Characteristics and Reflexivity

The research team comprised 4 investigators with complementary expertise: an engineer specializing in assistive technology with long-standing practice and research experience, a registered nurse contributing clinical insights and implementation competence, an implementation researcher with a track record in qualitative and quantitative studies of health care innovation and implementation, and a social psychologist with extensive practitioner- and research-based experience in organizational development processes.

All authors shared an interest in exploring the implementation of digital technologies in the health sector, which informed the study design and interpretation. None of the investigators had prior personal or professional relationships with the participants. To mitigate bias, they discussed their assumptions and interpretations during data analysis. This reflexive process helped ensure that findings were grounded in participants’ experiences rather than researchers’ preconceptions.

### Context

The study was conducted at an Assistive Technology Center serving approximately 300,000 residents in a mostly sparsely populated county in Central Sweden. The center is responsible for assessing needs, providing, maintaining, and repairing assistive products, including IoT-equipped powered wheelchairs. It operates under the Regional Authority, which oversees health care and assistive technology provision across the county. In addition, the Regional Authority is responsible for dental care, public health, public transport, research, culture, and, to some extent, education. To support these operations, the authority maintains a central IT department and an application board, which prepares and manages matters related to digital applications and their use within the organization.

At the time of the study, the Assistive Technology Center had recently integrated IoT-equipped powered wheelchairs into routine practice to support usage monitoring, simplify troubleshooting, and improve maintenance and service processes. Through mobile network connectivity, the IoT system enabled remote monitoring of parameters such as battery condition, system errors (eg, joystick-related faults), motor current, and the position and inclination of the seat and footrest. The system also provided access to geographic location in cases of theft or other emergencies. Automatic alerts supported early fault detection, diagnostics, service planning, and preventive maintenance by technicians. Approximately 60 IoT-equipped wheelchairs were in active use during the interview period.

### Recruitment and Participants

Participants were purposively selected to capture diverse experiences of how the IoT-equipped powered wheelchairs were implemented. First, the Assistive Technology Center provided the names of 2 staff members who had been engaged in the implementation process and who worked with IoT-equipped powered wheelchairs daily. During a process-mapping interview, they provided the names of 4 people who had played key roles during the implementation, representing different stakeholders. These 6 individuals were included based on their involvement in the process under study. The stakeholders and the participants’ roles are presented in Table 1.

**Table 1.** Stakeholders, participants’ roles and numbers, and interview durations.

Stakeholders	Participants’ roles and numbers	Interview durations (min)
Assistive Technology Center, Regional Authority	Technician, 2 males	51
Assistive Technology Center, Regional Authority	Head of Department, Mobility, 1 male	34
IT Department, Regional Authority	Head of Administration, 1 male	31
Application Board, Regional Authority	Administrative Manager, 1 female	26
Supplier of IoT^a^-equipped wheelchairs	Sales Development Manager, 1 male	27

a IoT: internet of things.

The participants were asked to recommend additional people to talk to, ensuring that we captured relevant people involved in the implementation process. One additional person was suggested, but when contacted, he said that he had not been involved in the process. Thus, we concluded that we had interviewed all key people involved in the implementation process. The sampling strategy was guided by the study’s focus on a specific implementation process rather than capturing the perspectives of all stakeholder groups affected by the technology. The participants were selected because they had direct responsibilities and active roles in the implementation of IoT-equipped powered wheelchairs. They were therefore considered information-rich sources regarding the events, decisions, facilitators, and barriers that shaped the process.

### Ethical Considerations

Based on their assessment that no sensitive personal data would be collected, the Swedish Ethics Review Authority dismissed the application for ethical review of the study, as the study does not involve sensitive personal data or physical interventions. The interviews were conducted after obtaining informed consent from the participants, who could withdraw from the study at any time without providing a reason. Data protection regulations were followed.

### Data Collection

Data were collected in 2 steps. In step 1, JB interviewed 2 participants in February 2024 to map the process and identify key stakeholders, with all information recorded in writing. In step 2, KP, ME, and JB collected data from 6 participants (including a second interview with the 2 participants from step 1) through semistructured interviews conducted between August 2024 and January 2025. Four individual online interviews and one pair-wise face-to-face interview were conducted and audio-recorded. The interview durations ranged from 26 to 51 minutes (Table 1), with a median time of 31 (IQR 27-34) minutes.

Based on the purpose and research questions of this study, the authors developed an interview guide for step 2, which included 5 main questions and 20 follow-up questions (Multimedia Appendix 1). The questions addressed issues such as the process, the stakeholders involved, and the barriers and facilitators to the implementation of IoT technology.

### Data Processing

Manually recorded data from step 1 were compiled to map the implementation process and prepare for step 2. The audio-recorded data from step 2 were transcribed using TurboScribe (Leif Erikson Ventures, LLC) [22]. The individual transcriptions were reviewed, corrected manually, and anonymized.

### Data Analysis

To address research questions 1 and 2, data from step 1 were compiled, and a preliminary process map indicating key stakeholders and major events was developed.

In the inductive, reflexive thematic analysis [23,24] of data from step 2, conducted to address research question 3, a hierarchical structure (subthemes→themes→meta-themes) was employed to represent progressively higher-order interpretations of patterned meaning across the dataset. This approach is consistent with multilevel thematic organization as described by Attride-Stirling [25]. The analysis followed six phases: (1) familiarization with the data, (2) generation of initial codes, (3) searching for themes, (4) reviewing themes, (5) defining and naming themes, and (6) producing the report. An example of the analysis process from meaning units (quotes) to meta-themes is provided in Multimedia Appendix 2.

In line with the opportunities for using ChatGPT in thematic analysis identified by Lee et al [26], GPT-5 was employed in the analysis. The analysis was conducted in Swedish to ensure that all interpretations were grounded in the interview data and that the extracted quotes could be verified against the source material. In phase 2, ChatGPT was used to generate codes representing facilitators and barriers from individual interviews and to illustrate each code with a corresponding quote. In phases 3 and 5, based on the codes from all interviews, ChatGPT generated themes and subthemes addressing research question 3. Each subtheme was supported by its underlying codes to allow verification. When presenting the themes, ChatGPT provided concise descriptions of their core ideas. Given the relatively large number of themes, ChatGPT was subsequently instructed to synthesize them at a higher level, resulting in three meta-themes. In phase 6, ChatGPT was used to translate illustrative quotes, summarize the meta-themes, and review the language in selected parts of the paper. The translated quotes were compared with the original quotations to ensure accuracy and were revised where necessary to better reflect their intended meaning. Details of the use of ChatGPT are provided in Multimedia Appendix 3.

As part of the inductive, reflective thematic analysis, the authors familiarized themselves with the interview transcripts to gain an understanding of the data. They reviewed the generated codes to ensure that they captured essential data. The authors reviewed and refined the subthemes, themes, and meta-themes to ensure comprehensive coverage of the data and to ensure that barriers and facilitators were thoroughly addressed. Quotations were used to illustrate each theme.

Finally, to address research question 4, the resulting meta-themes, themes, and subthemes were mapped onto the sociotechnical framework. Thus, the framework was applied analytically after the inductive thematic analysis and was not used deductively to generate the themes.

### Techniques to Enhance Trustworthiness

The authors engaged with the data to develop informed insights and employed rich, illustrative quotations to support the identified subthemes and themes, thereby enhancing the credibility of the findings. Although the study used a case study design, descriptions of the participant sample, research setting, and methodological procedures were provided to support transferability. Given the case study design and the lesser number of participants, the findings are not intended to be statistically generalizable. Rather, the study provides contextually grounded insights into one implementation process, which may offer analytical transferability to similar health care technology implementation contexts. The use of ChatGPT contributed to the trustworthiness of the analysis, particularly in terms of credibility and dependability, by offering objectivity during data familiarization, coding, theme refinement, and the selection of representative quotes. The coding process and theme development were systematically documented, and the coding was reviewed by the authors to enhance dependability.

## Results

### Events and Stakeholders in the Implementation Process

In the case studied, the implementation of IoT-equipped powered wheelchairs was initiated by the supplier. Although the technology had been integrated into their wheelchairs as early as 2019, it had not yet been used in Sweden. The supplier encouraged the Assistive Technology Center to begin using the technology. Some of the technicians at the Center realized that the technology could be useful, as they were responsible for the maintenance and repair of powered wheelchairs in a geographic area of nearly 30,000 square kilometers. The Assistive Technology Center brought the matter to the regional application board, which initially rejected the use of the technology due to concerns related to the General Data Protection Regulation (GDPR), including the ownership of the server where wheelchair data were stored, which was located outside the European Union and GPS data being classified as personal data. The regional application board also requested that 2-factor authentication be implemented. Following repeated communications between the Assistive Technology Center, the supplier, and the application board, the application board approved the use of the IoT technology. This decision was based on several changes: the data server was relocated to Sweden, the GPS functionality was disabled, and a form of 2-factor authentication was established. The latter was achieved by linking each user to their respective wheelchair using the serial number and information stored in the Assistive Technology Center’s digital assistive product management system, to which the supplier did not have access. While the supplier continued to collect GPS data, it did not share it with the Center except under specific circumstances, such as theft.

In parallel with discussions involving the regional application board, the Assistive Technology Center also engaged with the IT department of the regional authority responsible for health care. The use of the supplier’s software for managing IoT-equipped powered wheelchairs required technicians to have local administrator rights. These rights were initially restricted but were later permitted. After obtaining the necessary permissions, the Assistive Technology Center’s management team decided to deploy the IoT technology in 2023.

**Figure 2.**
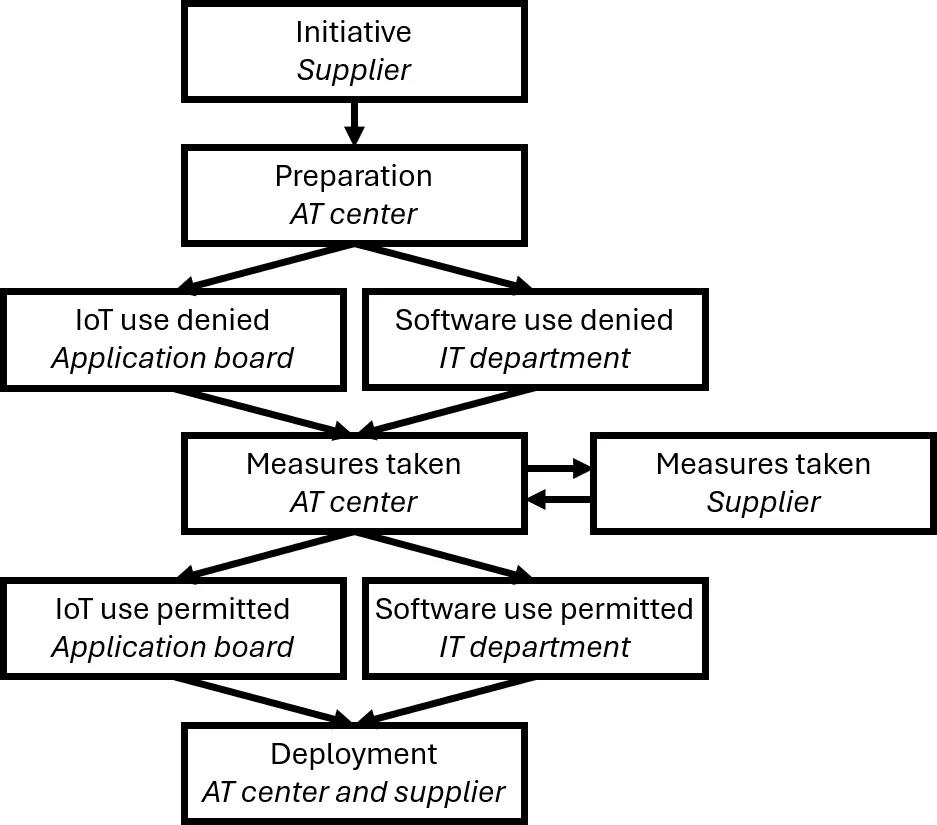
A preliminary process map of major events and stakeholders involved in the implementation process of the Internet of Things (IoT)–equipped powered wheelchairs. AT: assistive technology.

Based on the initial interviews with the technicians, a preliminary reconstruction of the sequence of the 9 major events and the 4 stakeholders involved in the process is outlined schematically in Figure 2. The boxes in the figure do not represent the exact number of events or interactions between stakeholders.

### Facilitators and Barriers in the Implementation Process

**Figure 3.**
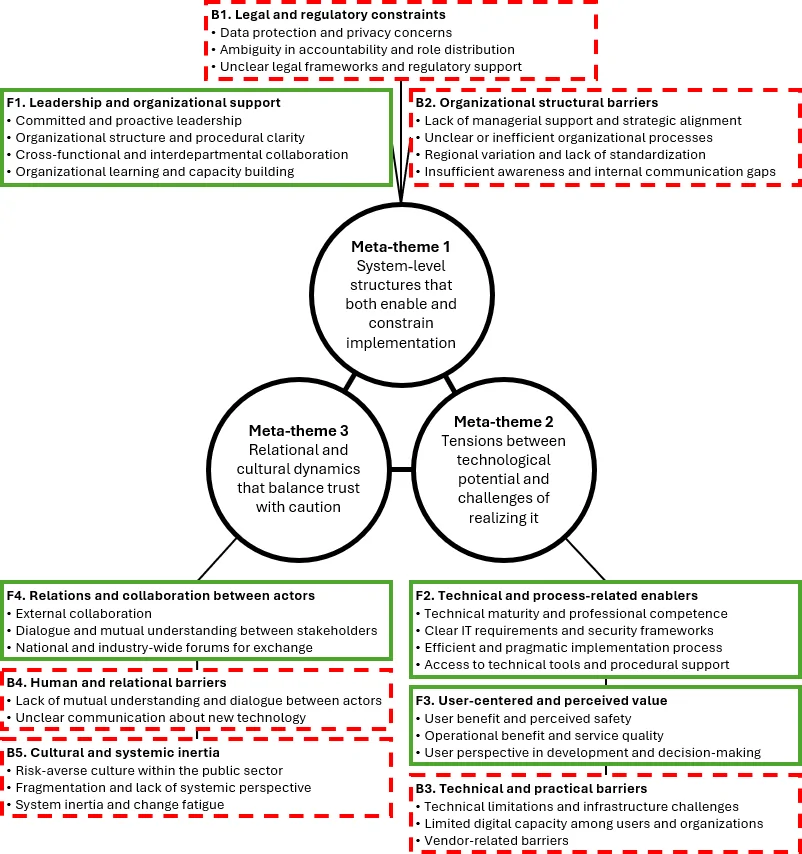
Meta-themes, themes, and subthemes of facilitators (F, solid green line) and barriers (B, dashed red line) in the implementation process of Internet of Things (IoT)–equipped powered wheelchairs.

The thematic analysis of the interview transcripts yielded 9 themes and 29 subthemes, which were organized into 3 meta-themes (Figure 3). These meta-themes represent overarching tensions and mechanisms that span both facilitators and barriers related to the implementation of IoT-equipped powered wheelchairs.

#### System-Level Structures That Both Enable and Constrain Implementation

The implementation process was largely shaped by the design of organizational and legal systems, including their processes, roles, and decision-making pathways.

##### Leadership and Organizational Support

The implementation of IoT-equipped powered wheelchairs was facilitated by committed and proactive leadership that demonstrated engagement and clear managerial anchoring. Supportive and competent colleagues within the organization further reinforced this process. In the words of 1 participant:

*We got a new manager who was interested in this. Because it’s important to have a manager who is interested... He said, yes, go ahead and keep this going*.

[Interview with Technicians, Assistive Technology Center]

Clear organizational structures and procedures also contributed to the implementation. Collaboration and structured decision-making processes involving multiple functions and departments ensured alignment across the organization. One participant described this approach as follows:

*We have a process for new and changed needs, where we look at the entire chain and involve operations, IT, and legal before making a decision*.

[Interview with Head of Administration, IT Department]

Over the course of the process, the stakeholder organizations developed and strengthened their capacity, which not only supported the implementation of the IoT-equipped wheelchairs but also generated valuable learning for future technology implementations.

##### Legal and Regulatory Constraints

Uncertainty about data protection, the division of responsibilities, and legislation created ambiguity and delays in the implementation process. Questions arose about whether the service complied with requirements under the GDPR and whether cloud solutions could be used for data storage. Concerns were also raised about privacy and the potential risk of data being accessed by actors in other countries.

Stakeholders experienced ambiguity in accountability and role distribution. The unclear allocation of responsibilities among stakeholders, including users themselves, was perceived as a barrier that contributed to delays. As 1 participant reflected:

*When are we, as municipality and region, responsible for the operation, and when is it the user themselves? It’s not always crystal clear*.

[Interview with Head of Administration, IT Department]

Unclear legal frameworks and insufficient regulatory support further complicated the process. Changes in the classification of health data, together with the absence of legislation enabling data exchange between stakeholders such as municipalities and regions, made implementation challenging.

##### Organizational and Structural Barriers

A lack of coordination and clear processes made the implementation effort cumbersome and vulnerable. Limited managerial engagement and strategic alignment were evident in the initial lack of driving leadership, the absence of a joint working group within the Assistive Technology Center, and insufficient national coordination. As one of the participants described:

*We were probably a bit resigned for a while because we didn’t think our manager drove this the way they should have*.

[Interview with Technicians, Assistive Technology Center]

Unclear or inefficient organizational processes further impeded progress. Participants described burdensome administrative routines, complex decision-making structures, and bureaucratic obstacles. At the same time, there was no established process for implementation, which contributed to uncertainty and inconsistency.

Regional variation and the absence of standardization added to the complexity. The supplier observed notable differences between Swedish regions, necessitating adaptations to the technical solution. Even within a single region, perceptions of the technology varied. Insufficient awareness and internal communication gaps also hindered implementation. This was reflected in the limited knowledge of the Application Board and in unclear communication channels within the region. This was illustrated by one participant, who explained:

*Partly, I can imagine that the operations don’t know that ... It may be somewhat unknown that there is an Application Board*.

[Interview with Administrative Manager, Application Board]

### Tensions Between Technological Potential and the Challenges of Realizing It

The studied implementation process revealed that the value of technology was realized only when stakeholders shared a common understanding of its benefits.

#### Technical and Process-Related Enablers

Practical, technical, and process-related factors created favorable conditions for the implementation and effective use of the IoT solution. Technical maturity and professional competence among stakeholders facilitated progress. This included the supplier’s improved ability to demonstrate the technology’s benefits, combined with key individuals’ understanding of the underlying problems. In addition, the local technicians were enabled to act as system administrators for the required software, which was not the case earlier.

Clear IT requirements and established security frameworks at both the organizational and international levels further supported the implementation. Updates to the Network and Information Systems Directive enabled compliance, while the region’s IT policies and information security classification guided the process. Additionally, the option to disable the GPS-tracking function of the IoT-equipped powered wheelchairs allowed adherence to security requirements.

An efficient and pragmatic implementation process was reflected in regular meetings, which strengthened communication between operations and IT, and a streamlined internal decision-making structure within the Assistive Technology Center. One participant put it in this way:

*We have just over a hundred employees, and with that also a management group that keeps track of most things and can steer and make it work as it should*.

[Interview with Head of Department, Mobility, Assistive Technology Center]

#### User-Centered and Perceived Value

Access to technical tools and procedural support contributed to a user-centered and perceived value for both the Assistive Technology Center and wheelchair users, which were recognized from the early stages of implementation. For users, perceived value was reflected in positive reactions to having their wheelchairs connected, including faster repairs and increased perceived safety, such as the ability to locate the wheelchair in the event of theft. From an operational perspective, the technology enabled earlier fault detection and planning of repairs, reduced the need to travel to investigate malfunctions, and allowed technicians to bring appropriate tools and parts, thereby saving a considerable number of working hours. According to 1 participant:

*We have found quite a lot of benefit with it when we’ve learned to handle error codes. And that we don’t need to travel long distances to investigate a fault.*

[Interview with Head of Department, Mobility, Assistive Technology Center]

These advantages were viewed as providing not only economic but also environmental value.

Through their close contact with wheelchair users, technicians brought forward users’ experiences and perspectives believed to influence the development and decision-making regarding IoT-equipped powered wheelchairs.

#### Technical and Practical Barriers

Technical barriers, insufficient adaptation, and low levels of digital maturity delayed or complicated the implementation process. Technical limitations and infrastructure challenges included the requirement to deactivate the GPS tracking function in the basic configuration and the inability to integrate cloud services. Additional issues involved firewalls that blocked software updates and uncertainty regarding data ownership.

Limited digital capacity among both users and organizations further constrained progress. Challenges included wheelchair users’ limited ability to manage digital technology, inadequate authentication procedures in applications, and a generally slow digital transition within the region. As 1 participant remarked:

*What you notice is this sluggish transformation into a digital society within the regions in general... they’re perhaps not always the quickest to act*.

[Interview with Sales Development Manager, Supplier]

Regarding the supplier, participants perceived a lack of understanding of data protection and highlighted the limited number of suppliers, which resulted in weak competition.

### Relational and Cultural Dynamics That Balance Trust With Caution

The studied implementation process was influenced by relational dynamics, such as trust, communication, and collaboration, both internally and externally.

#### Relations and Collaboration Among Stakeholders

Successful implementation depended on effective relationships and communication between regions, suppliers, and supporting functions. External collaboration was facilitated by good coordination between the Assistive Technology Center and the supplier. Participants also suggested that an industry association could play a more active role as a driving force in fostering collaboration between suppliers and regions. As one participant pointed out:

*I think an organization like [industry association] could be more of a driving force ... a forum where we suppliers, together with the regions, can discuss*.

[Interview with Sales Development Manager, Supplier]

External factors also influenced the implementation process. For example, a new agreement between the European Union and the United States eased approval procedures for certain cloud services.

Within the regions, implementation was facilitated by dialogue and mutual understanding among stakeholders, particularly among operational, legal, and IT functions. One participant illustrated this by saying:

*We started talking with legal: how do we solve this, then? If you say no, can we not do this? What is your thinking?*

[Interview with Administrative Manager, Application Board]

National and industry-wide forums for exchange were also mentioned as facilitators. These included seminars and networks where participants could learn how other regions addressed similar challenges. The industry association was likewise seen as a potential platform for dialogue and collaboration between suppliers and regions.

#### Human and Relational Barriers

Uncertainty and communication difficulties across organizational boundaries negatively affected the implementation. The lack of mutual understanding and dialogue among stakeholders was reflected in the limited involvement of wheelchair users, differing perspectives between IT and operational staff, and challenges in conveying needs to decision-makers. This was illustrated by 1 participant:

*The process itself has not really been difficult. The problem is getting in touch with all of these key stakeholders...[and] how to phrase the problem [for the Application Board]*.

[Interview with Technicians, Assistive Technology Center]

The limited user involvement also contributed to unclear communication about the new technology. As 1 participant reflected:

*Was there an opportunity for the users to participate? No, it was probably mostly us who decided that*.

[Interview with Head of Administration, IT Department]

#### Cultural and Systemic Inertia

Caution and fragmented systems made it difficult to introduce the new digital solution. A risk-averse culture within the public sector was reflected in the region’s slow transition toward digitalization and a prevailing tendency to focus on problems rather than possibilities.

*People on the IT security side more often tend to see this as a problem… Operations see more possibilities*.

[Interview with Administrative Manager, Application Board]

System inertia was further evident in the complex and time-consuming relationships among the region, the supplier, and the users. In addition, legal uncertainties made it challenging to obtain approval for digital solutions.

*[T]here have been uncertainties around GDPR and that whole area, which the service actually meets all the requirements for ... but toward the region, municipality, governing body, it isn’t exactly easy to get application solutions approved*.

[Interview with Sales Development Manager, Supplier]

At the national level, fragmentation and the absence of a systemic perspective resulted in regions procuring different solutions, which required suppliers to adapt to varying local conditions. At the organizational level, the process was perceived to have been easier if there had been a joint working group within the Assistive Technology Center.

### A Sociotechnical Perspective on the Implementation Process

The results concerning events and stakeholders in the implementation process illustrate the complex interplay of sociotechnical factors that affect IoT implementation processes in health care. The range of factors also highlights the importance of simultaneously addressing both the technical and social factors. From a sociotechnical systems perspective, the 5 key dimensions identified by Kronlid et al [9]: laws and regulations, organizational support, user focus, resources, and infrastructure, emerged as critical to both enabling and constraining the process of implementing IoT-equipped powered wheelchairs. The results underscore the importance of aligning several aspects of those dimensions. That is, it is not enough to address a single factor; only when multiple factors are tackled in parallel, and the solutions are viewed in relation to one another, does a systemic perspective emerge, making implementation possible.

Rather than being driven by isolated technical decisions, the implementation process was shaped by interdependent cultural, organizational, and infrastructural dynamics. The case reveals how implementation was made possible through negotiation among stakeholders embedded in complex structures—where legal ambiguity, fragmented governance, and uneven digital readiness interact with leadership, operational routines, and relational trust. Progress depended not only on resolving discrete challenges but also on aligning multiple layers of the system, from regulatory compliance and organizational capacity to infrastructural adaptability and user legitimacy. The uneven involvement of end users and the tension between operational pragmatism and systemic inertia further illustrate how sociotechnical change is contingent on the ability to coordinate across domains, bridge institutional silos, and cultivate shared meaning around technological value.

The 3 meta-themes can be understood in relation to the 5 dimensions of the sociotechnical framework proposed by Kronlid et al [9] and Palm et al [21]. Meta-theme 1 primarily reflects the dimensions of laws and regulations and organizational support, illustrating how regulatory requirements and organizational structures jointly enabled and constrained implementation. Meta-theme 2 mainly relates to infrastructure and resources, highlighting how technical capabilities depended on organizational competencies, support functions, and available resources. Meta-theme 3 cuts across all dimensions of the framework by emphasizing how trust, communication, collaboration, and cultural norms influenced how regulatory, organizational, technical, and resource-related conditions were interpreted and enacted in practice.

While the first 2 meta-themes broadly correspond to dimensions identified in the sociotechnical framework, the third meta-theme emerged inductively from the data. Rather than representing a separate system dimension, it may be understood as a cross-cutting mechanism that shapes interactions among existing sociotechnical dimensions during implementation.

## Discussion

### Summary of Findings

This study investigated the implementation of IoT-equipped powered wheelchairs by a Swedish public assistive technology provider from a sociotechnical perspective. The findings show that implementation is not a linear or purely technical process but a negotiated and iterative alignment process in which organizational structures, technological capabilities, and relational dynamics co-shaped what was possible in practice. The 3 meta-themes capture recurring tensions within and between these dimensions that require active coordination rather than one-time decisions.

### Tensions Within System-Level Structures

The first meta-theme underscores the dual role of system-level structures in shaping the implementation process. Formal organizational arrangements, legal frameworks, and decision-making pathways were central to both enabling and impeding the adoption of IoT-equipped powered wheelchairs. Consistent with sociotechnical theory, these structures functioned not merely as background conditions but as active components that interacted with human agency and technological capabilities [11]. Importantly, legal and organizational dimensions did not operate independently. Regulatory uncertainty increased the need for organizational coordination, leadership, and cross-functional dialogue, while stronger organizational support helped transform legal constraints from implementation barriers into manageable design requirements. The interaction between these dimensions, therefore, shaped not only whether implementation progressed but also how the technology was ultimately configured and used.

On the enabling side, committed leadership, managerial anchoring, and structured decision-making processes created legitimacy and momentum for implementation. Supportive leadership enabled collaboration across departments and helped translate operational needs into decisions at higher administrative levels. This finding aligns with previous research emphasizing leadership and organizational support as critical for successful digital health implementation [14].

At the same time, legal and regulatory frameworks, particularly data protection legislation, introduced uncertainty and delays. This has been observed in other studies, where regulatory ambiguity and fragmented governance complicate implementation [9]. Initial interpretations of GDPR-related requirements led to early rejection of the IoT solution by the regional application board. Importantly, however, the findings indicate that these regulatory challenges were not static barriers but evolved. A key development in the process was the shift in how legal expertise was engaged. In the early phase, legal stakeholders were approached primarily as gatekeepers tasked with answering whether the solution was permissible. In this configuration, a single negative assessment effectively halted progress.

Later in the process, this interactional pattern changed. Legal stakeholders were invited into dialogue and encouraged to engage in problem-solving discussions focused on how the technology could be implemented in a legally compliant manner. This shift allowed regulatory requirements to be translated into concrete technical and organizational adaptations, such as relocating data servers, disabling GPS functionality, and developing alternative authentication solutions. From a sociotechnical perspective, this illustrates how legal frameworks constrain or enable implementation depending on how they are operationalized within organizational processes. Beyond legal compliance, the findings highlight the importance of robust patient data protection and cybersecurity measures when implementing IoT-enabled health care technologies. Connected assistive products generate and transmit data that may reveal sensitive information about users’ health status, location, and daily activities, making secure data storage, access control, authentication, and continuous risk assessment essential. The adaptations made in this case, including the relocation of data servers, restrictions on GPS data access, and strengthened authentication procedures, illustrate how compliance with health data regulations and cybersecurity requirements can shape implementation decisions and influence stakeholder trust.

Organizational and structural barriers further amplified these tensions. The lack of standardized implementation processes and bureaucratic complexity made progress dependent on individual initiative rather than systemic support. This misalignment between organizational structures and operational practices highlights how system-level arrangements can undermine the realization of technological benefits if they are not adapted to support innovation.

### Tensions Between Technological Potential and Its Realization

The second meta-theme captures the tension between the recognized potential of IoT technology and the practical challenges of integrating it into everyday operations. Stakeholders perceived clear benefits, including proactive maintenance, improved troubleshooting, reduced travel, and increased safety for wheelchair users. These anticipated gains are consistent with the broader literature on IoT in health care [7].

Realizing these benefits, however, depended on several enabling conditions. Technical maturity, professional competence, and the ability to adapt systems pragmatically were crucial. Allowing technicians to function as local system administrators and selectively disabling certain functionalities exemplify how adaptations helped bridge the gap between system requirements and operational needs. These findings reinforce sociotechnical research, showing that successful implementation often relies on local workarounds and adaptive practices rather than strict adherence to predefined technical models [15].

At the same time, technical and practical barriers persisted. Infrastructure constraints, limited system integration, and uneven levels of digital maturity among organizations and users delayed implementation and limited functionality. These challenges illustrate that technological readiness alone is insufficient; infrastructural compatibility, organizational capacity, and user capabilities are equally important. This is in line with previous studies, which indicate that technological readiness alone is insufficient and that infrastructural compatibility and user capabilities are equally important [16].

Perceived value played a critical role in sustaining engagement with the technology. User-centered benefits, such as faster repairs and increased perceived safety, along with operational efficiencies, contributed to acceptance and commitment. This highlights the importance of aligning technological solutions with the values and needs of both users and service providers.

Similarly, infrastructure and resources were closely intertwined. Technical infrastructure alone was insufficient to support implementation; its successful use depended on staff competence, available time, managerial support, and access to appropriate support structures. Resource limitations, therefore, influenced how infrastructural capabilities could be used in practice.

### Tensions in Relational and Cultural Dynamics

The third meta-theme emphasizes the role of relational and cultural dynamics in shaping the implementation process. Successful implementation depended on effective collaboration and communication among multiple stakeholders, including operational staff, legal experts, IT functions, and suppliers. Dialogue and mutual understanding were essential for addressing uncertainty and reconciling differing perspectives on risk and opportunity. Collaboration with suppliers and participation in national or industry-wide forums facilitated knowledge exchange and reduced isolation, supporting findings from prior research that emphasize the role of networks in innovation diffusion [11].

A particularly important relational dynamic concerned the interaction between operational units and legal functions. When legal stakeholders were positioned primarily as external reviewers, implementation stalled. When they became collaborative partners, implementation progressed. This transition reflects a broader cultural shift from a risk-averse, compliance-focused mindset toward a more solution-oriented approach, where legal expertise was mobilized to enable innovation within regulatory boundaries rather than to block it [27].

Despite these advances, relational barriers remained. Limited user involvement, challenges in communicating operational needs to decision-makers, and differences in professional perspectives hindered a shared understanding [28]. This finding resonates with research describing a risk-averse culture in public-sector organizations, where concerns about accountability, regulatory compliance, information security, and potential negative consequences can lead decision-makers to prioritize caution over experimentation [29]. In our case, these dynamics were reflected in lengthy approval processes related to GDPR compliance, differing risk perceptions between operational and IT/security actors, and the slow pace of digital transformation within the region. This caution should not be interpreted simply as resistance to innovation; rather, it can be understood as an attempt to balance technological opportunities with responsibilities related to safety, privacy, legal compliance, and public accountability. This suggests that the successful implementation of IoT-equipped assistive products depends not only on demonstrating technical value but also on establishing organizational legitimacy and trust across stakeholder groups.

From a sociotechnical perspective, these findings illustrate that trust, dialogue, and shared sense-making are not peripheral but central to technological implementation. The ability to move from gatekeeping to cocreation depended on leadership support, relational trust, and organizational willingness to tolerate uncertainty during the implementation process [9,30]. The findings suggest that overcoming such inertia requires cultural change, supported by leadership, shared learning, and trust-building across and within organizations.

Rather than constituting an independent sociotechnical dimension, the relational and cultural dynamics identified in this study appeared to influence how the other dimensions were enacted. Trust, communication, and shared understanding shaped how stakeholders interpreted regulations, mobilized organizational support, used resources, and engaged with technological infrastructure.

### Implications From a Sociotechnical Perspective

Taken together, the findings underscore that the implementation of IoT-equipped powered wheelchairs constitutes a sociotechnical transformation rather than a discrete technological intervention. Progress depended on aligning legal, organizational, technical, and relational factors simultaneously. Legal stakeholders emerged not merely as part of the institutional context but as active participants within the sociotechnical system, and their role and positioning significantly influenced implementation outcomes [9].

The study contributes to the limited empirical literature on IoT implementation in the assistive technology sector by demonstrating how facilitators and barriers are interdependent and evolve over time. The findings suggest that future implementations should adopt holistic strategies that integrate regulatory interpretation, organizational capacity-building, technological adaptation, and relational coordination. Such approaches may help organizations navigate tensions between innovation and accountability while enabling sustainable adoption of IoT-enabled assistive products.

These findings suggest that implementation capacity in public assistive technology services depends on how governance and regulatory requirements are translated into everyday work, how technical adaptations are negotiated, and how collaboration and trust are built across functions and levels.

### Policy Implications

The findings suggest 3 priority areas for policy and practice. First, uncertainty regarding data protection requirements, responsibility distribution, and regulatory compliance was a major implementation barrier. Policymakers and health care organizations should therefore develop clearer guidance regarding data governance, responsibility allocation, and GDPR compliance for IoT-enabled assistive products. Second, successful implementation depended on collaboration between operational staff, legal experts, and IT functions. Health care organizations should establish formal cross-functional implementation teams early in the implementation process to support joint problem-solving and reduce delays caused by fragmented decision-making. Third, regional variation and the lack of standardized approaches created additional complexity for both health care organizations and suppliers. Greater coordination across regions, including shared implementation procedures and common assessment criteria for connected assistive products, could reduce duplication of effort and facilitate wider adoption.

### Limitations

The study has some limitations that should be considered when interpreting the findings. First, the findings rely on participants’ retrospective accounts, which may have been influenced by recall bias or social desirability. To mitigate this risk, the analysis was grounded in verbatim interview transcripts, and illustrative quotations were used to support the identified themes.

Second, the study focused on stakeholders directly involved in the implementation process, from initiation to the deployment decision, and did not include wheelchair users, frontline clinicians such as occupational therapists and physiotherapists, or policymakers beyond those participating in the study. These groups may hold perspectives that differ from those reported here, particularly regarding user experience, clinical integration, and broader policy considerations. Therefore, the findings should be interpreted as an account of the implementation process from the perspective of actors involved in its planning, governance, and execution, rather than as a comprehensive representation of all stakeholders affected by the technology.

Third, as the study was conducted within a single Assistive Technology Center in Sweden, direct transferability to other health care systems or countries may be limited, particularly where organizational structures, regulatory frameworks, reimbursement systems, technical infrastructures, or roles in assistive technology provision differ. However, some identified implementation factors may be relevant to similar contexts involving connected assistive products and digitally enabled services. To support assessments of transferability, descriptions of the study context, participants, and methods have been provided.

### Conclusions

This study contributes to the emerging literature on the implementation of IoT-enabled assistive products by demonstrating how implementation unfolds as a sociotechnical process shaped by interactions among regulatory, organizational, technical, and relational factors. Rather than acting independently, these factors influenced one another throughout the implementation process, highlighting the importance of understanding technology adoption as a process of system alignment rather than technology deployment alone.

A key contribution of the study is the identification of relational and cultural dynamics, including trust, communication, and cross-functional collaboration, as mechanisms that shape how existing sociotechnical dimensions are interpreted and enacted in practice. This suggests that successful implementation depends not only on technological readiness and regulatory compliance but also on the ability of stakeholders to negotiate shared understandings and coordinate action across organizational boundaries.

Although the study was conducted in a single Swedish assistive technology service context, the findings provide transferable lessons for other public organizations implementing connected assistive products and digital health solutions. In particular, the study highlights the importance of early engagement between operational, legal, and IT functions, clear approaches to data governance, and organizational structures that support collaboration across professional and institutional boundaries.

## Supplementary material

10.2196/92414 Multimedia Appendix 1 Interview guide.

10.2196/92414 Multimedia Appendix 2 An example of the analysis process.

10.2196/92414 Multimedia Appendix 3 Details of the use of ChatGPT in the analysis.

## Abbreviations

GDPR: General Data Protection Regulation
IoT: Internet of Things
