# Supplementary material for: Stakeholder Perspectives on Facilitators of and Barriers to the Implementation of Internet of Things–Equipped Powered Wheelchairs in Sweden: Qualitative Case Study
Source: JMIR Rehabil Assist Technol. 2026 Aug 28;13:e92414. doi: 10.2196/92414 (PMC13524362; doi:10.2196/92414)
Supplement: Supplementary file 1 [file rehab-v13-e92414-s001.docx]

## Multimedia Appendix 1. Interview guide

Based on the purpose and research questions of this study, the authors developed the following interview guide for Step 2.

| *Main questions* | *Follow-up Questions* (**Bold** = High priority*; Italics* = Low priority) |
| --- | --- |
| Can you describe the **process** of implementing IoT technology to support the service and maintenance of electric wheelchairs? | 1. **Which stakeholders were involved in the process and in the decisions that were made?** 2. **What opportunities existed for other stakeholders, including users of electric wheelchairs, to be involved in the process?** 3. **What role did you and your department/organisation have during the introduction?** 4. **Which decisions were central to make, and how were they made?** 5. **How do you think the process worked?** 6. Were needs and challenges identified before decisions were made? [If yes] What specific steps were taken to identify needs and challenges in the service and maintenance of electric wheelchairs before considering the IoT solution? 7. Were different solutions discussed? [If yes] How were different IoT solutions assessed and selected to meet the needs within the service and maintenance of electric wheelchairs? |
| Can you describe the **organisational structures** involved in implementing IoT technology to support service and maintenance of electric wheelchairs? | 8. Did the organisational structure need to be adapted to support the introduction? [If yes] How was it adapted? 9. Are you aware of any organisational structures that facilitated the introduction? [If yes] What were they, and how were they used? 10. Are you aware of any organisational structures that hindered the introduction? [If yes] What were they, and how did you handle them? |
| Can you describe **guidelines or regulations** that were relevant for introducing IoT technology to support service and maintenance of electric wheelchairs? | 11. Do you feel familiar with the guidelines and regulations governing the possibility of introducing the technology? [If yes] Which guidelines and regulations were relevant to the introduction? 12. Were there guidelines or regulations that facilitated the introduction? [If yes] How were they used? 13. Were there guidelines or regulations that hindered the introduction? [If yes] How were these obstacles handled? |
| Can you describe your **additional impressions** on the process of introducing IoT technology to support the service and maintenance of electric wheelchairs, and how it has worked? | 14. **Were there other factors besides organisational structures, guidelines, and regulations that influenced the introduction? [If yes] What were they, and how did they influence the introduction?** 15. **What would you or your department/organisation have wanted to do differently?** 16. **What prevented you from doing things differently?** 17. **What could others have done differently?** |
| Can you describe the **expectations** attached to the introduction of IoT technology to support the service and maintenance of electric wheelchairs, and the extent to which they have been met? | 18. *Was there anything you hoped IoT on electric wheelchairs would contribute? [If yes] What was it, and was it achieved?* 19. *Is the effectiveness and usefulness of the technology being evaluated? [If yes] How is effectiveness and usefulness evaluated? [If no] Why not?* 20. *Was user acceptance and training related to use discussed? [If yes] What measures were taken to ensure user acceptance and training related to the use of IoT on electric wheelchairs?* |
