# Supplementary material for: Stakeholder Perspectives on Facilitators of and Barriers to the Implementation of Internet of Things–Equipped Powered Wheelchairs in Sweden: Qualitative Case Study
Source: JMIR Rehabil Assist Technol. 2026 Aug 28;13:e92414. doi: 10.2196/92414 (PMC13524362; doi:10.2196/92414)
Supplement: Supplementary file 2 [file rehab-v13-e92414-s002.docx]

## Multimedia Appendix 2. An example of the analysis process

| **Meta-theme** | **Theme** | **Sub-theme** | **Code** | **Meaning unit** | **Participant** |
| --- | --- | --- | --- | --- | --- |
| **System-level structures that both enable and constrain innovation** | **Leadership and organizational support** | **Committed and proactive leadership** | Driving leadership within the organization | As driving forces, we’ve tried to demonstrate that new technology is coming. It’s not just about the technology — it’s also our way of working that needs to develop. | Head of Department, Mobility |
|  |  |  | Supportive colleagues and internal competence | We had [name] employed… He was on our side and also wanted to drive this forward. | Technicians |
|  |  | **Organizational learning and capacity building** | Learning process for future implementations | So the next time we do it, we know where the difficulties were. So we can perhaps address them right away. | Sales Development Manager |
|  |  |  | Environmental scanning and learning | I do some environmental scanning through seminars and networks, so we’re constantly learning how other regions have solved similar issues. | Head of Administration |
|  | **Legal and regulatory constraints** | **Data protection and privacy concerns** | GDPR and legal uncertainty | …there have been uncertainties around GDPR and that whole area, which the service actually meets all the requirements for … but toward the region, municipality, governing body, it isn’t exactly easy to get application solutions approved. | Sales Development Manager |
|  |  |  | Uncertainty around cloud storage and data access | The reason this implementation decision took time was that it was unclear whether it was legal to store data in the cloud. | Head of Administration |
|  |  | **Ambiguity in accountability and role distribution** | Unclear allocation of responsibility | A great deal of responsibility rests on us … but then, really, it’s the regions that bear the responsibility toward the user … we are a chain of responsible actors. | Sales Development Manager |
|  |  |  | Unclear boundary between regional and user responsibility | When are we, as municipality and region, responsible for the operation, and when is it the user themselves? It’s not always crystal clear. | Head of Administration |
