# Supplementary material for: Stakeholder Perspectives on Facilitators of and Barriers to the Implementation of Internet of Things–Equipped Powered Wheelchairs in Sweden: Qualitative Case Study
Source: JMIR Rehabil Assist Technol. 2026 Aug 28;13:e92414. doi: 10.2196/92414 (PMC13524362; doi:10.2196/92414)
Supplement: Supplementary file 3 [file rehab-v13-e92414-s003.docx]

## Multimedia Appendix 3. Details of the use of ChatGPT in the analysis

Details of the use of ChatGPT in the first five phases of the thematic analysis of the interviews are provided in the following table

| **ChatGPT use** | **Output** | **Comment** |
| --- | --- | --- |
| *Phase 1 Familiarization with the data* | | |
| None |  |  |
| *Phase 2 Generation of initial codes* | | |
| Prompt: The attached file contains a transcript of an interview in Swedish. The purpose of the interview was to answer the research question: What facilitators and barriers do stakeholders perceive as influencing the implementation of Internet-of-Things-equipped powered wheelchairs (sometimes referred to as [name])? Please list codes in Swedish as is done in thematic analysis and a quote illustrating each code. | Codes and illustrative quotations for each code, categorized into facilitators and barriers. | This prompt was repeated for each of the five interviews. For each interview, 8 to 14 codes in Swedish were generated for facilitators and barriers, respectively.  For each interview, ChatGPT was prompted to prepare an Excel file with the code, quotation, and category (facilitator or barrier). Later, ChatGPT was asked to prepare a bilingual Excel file where the codes and quotations were translated into English. These translations were checked by author JB. |
| *Phase 3 Searching for themes* | | |
| Prompt: Now I want you, based on the codes for facilitators from the five analysed interviews, to develop themes and subthemes for facilitators, as is done in thematic analysis, and to link the different facilitator codes to their respective themes and subthemes. | Four themes were generated, each with 3 or 4 subthemes. | ChatGPT was prompted to prepare an Excel file with the theme, sub-theme and codes. |
| Prompt: Now I want you, based on the codes for barriers from the five analysed interviews, to develop themes and subthemes for barriers, as is done in thematic analysis, and to link the different barrier codes to their respective themes and subthemes. | Five themes were generated, each with 3 or 4 subthemes. | ChatGPT was prompted to prepare an Excel file with the theme, sub-theme and codes. |
| Prompt: Is it possible to create themes at an even higher level by combining the total of nine overarching themes for facilitators and barriers? What are your thoughts on such a synthesis from the perspective of Braun and Clarke? | ChatGPT explained that it is possible to create meta-themes referencing [24, 25]  ChatGPT generated 3 meta-themes in Swedish: System governance and structure; Relationships, collaboration, and culture; and Technological opportunities and perceived usefulness. | Given this information, the literature was searched for methods related to creating meta-themes. Besides [24, 25], [26] was used as a methodological basis. |
| Prompt: Please translate all meta-themes, themes and subthemes into academic American English.  Prompt used later: Translate the subthemes. | ChatGPT provided translations of all meta-themes (System-level structures that both enable and constrain innovation; Relational and cultural dynamics balancing trust and caution; and The tension between technological potential and the challenges of realizing it), themes (Organizational leadership and support; Regulatory and procedural frameworks; Tension between control and flexibility; Cross-functional and inter-organizational collaboration; Communication, trust, and cultural change; Tension between trust and caution; Technical maturity and competence; User-centered value and perceived benefits; and Tension between potential and complexity), and subthemes. | As ChatGPT did not translate the subthemes along with the meta-themes and the themes, ChatGPT was asked again to do it on a later occasion. |
| *Phase 4 Reviewing themes* | | |
| As the English translations of the themes did not correspond well with the original Swedish names, ChatGPT was asked to translate the original Swedish names. ChatGPT suggested a harmonized phrasing for all nine themes. | ChatGPT provided the following harmonized names of the themes in English: Leadership and organizational support as enablers; Technical and process-related enablers; Relational and inter-organizational enablers; User-centered and value-based enablers; Legal and regulatory barriers; Organizational and structural barriers; Technical and practical barriers; Human and relational barriers; and Cultural and systemic barriers.  ChatGPT also suggested optional condensed wording: Leadership and organizational support; Technical and process-related enablers; Relational and inter-organizational collaboration; User-centered value and perceived benefit; Legal and regulatory barriers; Organizational and structural barriers; Technical and practical barriers; Human and relational barriers; and Cultural and systemic barriers. | The names of the meta-themes, themes and subthemes were reviewed by the authors.  A sub-theme in Human and relational barriers, named Resistance to change and perceived risk, was omitted as the interview material did not provide sufficient support. |
| *Phase 5 Defining and naming themes* | | |
|  |  | After reviewing the meta-themes, themes and subthemes, the authors selected the names listed below.  *Meta-themes*  System-level structures that both enable and constrain implementation; Tensions between technological potential and challenges of realizing it; and Relational and cultural dynamics that balance trust with caution.  *Themes*  Leadership and organizational support; Technical and process-related enablers; Relations and collaboration between actors; User-centered and perceived value; Legal and regulatory constraints; Organizational and structural barriers; Technical and practical barriers; Human and relational barriers; and Cultural and systemic inertia.  *Subthemes*  The English translations of the subtheme names suggested by ChatGPT were used. |
